# Cancer Cell-Intrinsic Type I Interferon Signaling Promotes Antitumor Immunity in Head and Neck Squamous Cell Carcinoma

**DOI:** 10.3390/cancers17081279

**Published:** 2025-04-10

**Authors:** Guiqin Xie, Cuicui Yang, Xiaowu Pang, Tzyy-Choou Wu, Xinbin Gu

**Affiliations:** 1 Department of Oral Pathology, Howard University, 600 W Street NW, Washington, DC 20059, USA; cuicui.yang@howard.edu (C.Y.); xpang@howard.edu (X.P.); 2 Cancer Center, Howard University, 2041 Georgia Avenue NW, Washington, DC 20059, USA; 3 Pathology, Oncology, Obstetrics & Gynecology, and Molecular Microbiology & Immunology, Johns Hopkins University School of Medicine, Baltimore, MD 21287, USA; wutc@jhmi.edu

**Keywords:** head and neck cancer, HNSCC, innate immunity, type I interferon, dendritic cell

## Abstract

**Background**: The cyclic GMP-AMP synthase (cGAS)–type I interferon (IFN-I) pathway detects cytoplasmic DNA and triggers immune responses. Cancer cells often suppress this pathway to evade immune surveillance; however, its therapeutic potential remains unclear. **Methods**: Mouse oral squamous cell carcinoma models, representing a prominent subtype of head and neck squamous cell carcinoma (HNSCC), were employed in this study. Flow cytometry, Western blot, ELISA, and PCR were used for analysis. **Results**: We found that immune-unresponsive MOC2 tumors exhibited a deficiency of antigen-presenting cells and cytotoxic T lymphocytes, along with a significant suppression of the cGAS-IFN-I pathway, compared to immune-responsive MOC1 tumors. An MOC2-conditioned medium impaired the differentiation of bone marrow-derived cells into dendritic cells (DCs), reducing the expression of DC markers as well as class I and II major histocompatibility complex (MHC) molecules. The activation of the cGAS-IFN-I pathway in MOC2 cells, either through exogenous DNA or direct IFN-I expression, enhanced class I MHC expression and antigen presentation on MOC2 cells. Furthermore, IFNB1 expression in MOC2 cells induced apoptosis and upregulated chemokines, such as CXCL9 and CXCL10, which recruit immune cells. In immunocompetent mice, IFNB1 expression suppressed MOC2 tumor growth by attracting DCs and T cells, an effect amplified by co-expressing the granulocyte–macrophage colony-stimulating factor. **Conclusions**: These findings highlight the potential of enhancing cancer cell-intrinsic cGAS-IFN-I signaling to improve tumor immune surveillance and control the progression of immune-cold HNSCC tumors.

## Simple Summary

This study investigates the role of the tumor cell–intrinsic cGAS–IFN-I pathway in immune surveillance using mouse models of oral squamous cell carcinoma, a major subtype of head and neck squamous cell carcinoma (HNSCC). Compared to immune-responsive MOC1 tumors, immune-resistant MOC2 tumors showed suppression of this pathway and lacked antigen-presenting cells and cytotoxic T cells. MOC2-conditioned medium impaired dendritic cell differentiation and reduced MHC molecule expression. The activation of the cGAS-IFN-I pathway in MOC2 cells increased antigen presentation, induced apoptosis, and elevated chemokine expression to recruit immune cells. Expressing IFNB1 in MOC2 tumors suppressed tumor growth by attracting dendritic cells and T cells, and this effect was further amplified by co-expressing GM-CSF, which promotes immune cell development. These findings suggest that boosting tumor cell–intrinsic IFN-I signaling may enhance antitumor immunity and help control immune-cold HNSCC.

## 1. Introduction

Head and neck squamous cell carcinoma (HNSCC) accounts for >500,000 new cases globally and ~37,000 new cases in the United States every year [1,2]. HNSCC comprises malignancies arising from the mucosal squamous epithelium lining various tissues of the head and neck, including the oral cavity, oropharynx, hypopharynx, nasopharynx, larynx, lips, and nasal cavity. Among these, oral squamous cell carcinoma (OSCC) is one of the most prevalent subtypes. The traditional treatments, ranging from surgery to chemo- and radiotherapy, are often associated with significant morbidity in HNSCC patients. The FDA-approved immune checkpoint blockade (ICB) to activate antitumor immunity has altered the landscape of HNSCC therapy [3] and achieved a durable clinical response in some patients. However, despite the promise of ICB in clinical settings, it remains ineffective for over 80% of HNSCC patients [4,5]. In ICB-refractory cancer patients, the limited presence of CD8^+^ T cells poses a major hurdle to immunotherapy efficacy [6]. Other immunotherapies, such as CAR-T therapy and bispecific antibodies, have been investigated for HNSCC; however, their efficacy is still being evaluated in preclinical or clinical trials [4,7]. This challenge underscores the urgent need for research into the mechanisms of immune evasion in HNSCC tumors and the development of strategies to enhance antitumor immunity, ultimately improving patient survival and quality of life.

The cyclic GMP-AMP synthase (cGAS)–type I interferon (IFN-I) pathway detects cytoplasmic DNA, which typically signals infection and triggers immune defenses against viruses and bacteria. In cancer cells [8,9], cytoplasmic DNA can arise from genomic instability, DNA damage, or cell death due to uncontrolled tumor growth and stress—hallmarks of cancer. This cytoplasmic DNA may activate the cGAS pathway, leading to IFN-I production that enhances antigen presentation and stimulates cytokine production [10], both of which are essential for an antitumor immune response. cGAS is a pivotal DNA sensor in the innate immune pathway, detecting abnormal cytosolic DNAs and activating IFN-I via the stimulator of interferon genes (STING) in both cancer and immune cells. Emerging evidence highlights the critical role of cGAS-IFN-I-mediated DNA sensing in cancer cells for initiating robust immune responses. Notably, the activation of this pathway in cancer cells facilitates antigen cross-presentation and T-cell responses by enabling the transfer of cGAS-STING signaling from cancer cells to antigen-presenting cells (APCs) [11]. This transfer has been demonstrated as essential for generating durable antitumor immune responses [12]. Furthermore, the downregulation of interferons and interferon-stimulated genes (ISGs) in established tumors has been linked to dysfunctional IFN-I signaling and tumor progression [13]. In fact, studies have shown that the DNA-sensing defense response is one of the most inhibited pathways in immunotherapy-resistant HNSCC cells [14].

With the increasing prominence of immunotherapies against HNSCC, syngeneic tumor models in immunocompetent mice have been extensively used to investigate mechanisms of resistance. Among these, MOC1 and MOC2, murine cell lines derived from carcinogen-induced OSCC, are widely used to study HNSCC [15,16,17]. MOC1 forms localized tumors with slow growth, and MOC2 generates aggressive, poorly differentiated tumors with rapid growth and metastasis. MOC1 elicits a stronger immune response, making it useful for testing immunotherapies in early-stage tumors, while MOC2 exhibits immune evasion, serving as a model for studying immune escape and resistance in advanced cancers. For example, in mouse models, inflamed MOC1 tumors, which are sensitive to ICB treatment, exhibit strong responses to combined STING agonist and ICB treatment, resulting in protective immunity; in contrast, immune-cold MOC2 tumors fail to respond to the same regimen [18], indicating an impaired DNA sensing pathway in these immunotherapy-resistant cancer cells. Additionally, STING knockout in HNSCC cells has been shown to disrupt the crosstalk between natural killer cells and dendritic cells (DCs) in an in vitro setting [19], further underscoring the importance of the cancer cell-initiated cGAS-IFN-I signaling in inducing favorable antitumor immune responses. However, the mechanisms by which the impairment of this pathway in HNSCC enables immune evasion, as well as the strategies required to effectively stimulate it for robust antitumor immunity, remain largely unresolved.

In this study, we hypothesized that a dysfunctional cGAS-IFN-I DNA sensing pathway in HNSCC cells contributes to impaired DC function, thus reducing antitumor T-cell immunity. To test this hypothesis, we utilized the well-established MOC1 and MOC2 models in immunocompetent C57BL/6 mice to investigate the role of cancer-intrinsic IFN-I signaling in antitumor immunity. Our results revealed that the cGAS-IFN-I pathway was significantly suppressed in the more aggressive MOC2 tumors compared to MOC1 tumors. MOC2 cells inhibited the differentiation of bone marrow-derived cells into functional DCs in vitro and formed tumors with a marked deficiency of DCs within the tumor microenvironment (TME) compared to ICB-sensitive MOC1 tumors. Stimulating the cGAS-IFN-I pathway in MOC2 cells promoted DC recruitment to MOC2 tumors and induced robust antitumor T-cell responses. Notably, the co-expression of granulocyte–macrophage colony-stimulating factor (GM-CSF) and IFN-I within MOC2 tumors accelerated tumor regression, underscoring that IFN-I signaling in cancer cells supported DC function to drive antitumor immunity. These findings suggest that augmenting cancer cell-intrinsic IFN-I signaling may represent a promising therapeutic strategy for controlling the progression of “immune-cold” HNSCC.

## 2. Materials and Methods

### 2.1. Cell Cultures

MOC1 (#EWL001-FP) and MOC2 (#EWL002-FP) cells were purchased from Kerafast (Boston, MA, USA) with the company’s authentication certificates. The MOC2-derived sublines, control mKate2-MOC2 (MOC2^Ctl^) and mKate2-SIIN-MOC2 (MOC2^SIIN^) cells, were kindly provided by Dr. Clint Allen (NIH/NCI) [20,21]. A 293FT cell line was purchased from Thermo Fisher Scientific (#R70007; Waltham, MA, USA). The MOC1 and MOC2 murine cells and sublines were cultured in a 2:1 mixture of HyClone™ Iscove’s Modified Dulbecco’s Medium (IMDM) (#SH30228FS, Fisher Scientific, Pittsburgh, PA, USA) and HyClone Ham’s Nutrient Mixtures F12 (#SH3002601, Fisher Scientific), supplemented with 5% fetal bovine serum (FBS; #A5256701, Thermo Fisher Scientific), 1% antibiotic–antimycotic solution (#15140-122, Thermo Fisher Scientific), 0.1% insulin (5 mg/mL) (#I6634, Millipore Sigma, Rockville, MD, USA), hydrocortisone (40 µg/L) (#H0135, Millipore Sigma), and EGF (5 µg/L) (#2028-EG, R&D Systems, Minneapolis, MN, USA) (complete IMDM/F12 media), following the manufacturer’s instructions. 293FT cells were cultured in DMEM (#A4192101, Thermo Fisher Scientific), supplemented with 10% FBS and 1% antibiotic–antimycotic solution. Mouse bone marrow cells were cultured in complete RPMI medium (#11875-093, Thermo Fisher Scientific) containing 10% FBS and 1% antibiotic–antimycotic solution, supplemented with 5 ng/mL GM-CSF (#576304, Biolegend, San Diego, CA, USA) (bone marrow cell culture medium). Cells were maintained in a humidified incubator at 37 °C with 5% CO_2_.

### 2.2. Viral Transduction of Murine Cancer Cells

The murine *Csf2 (Gmcsf)* expression vector pMP71-GM-CSF (#174596), murine *Ifnb1* expressing vector pMP71-IFN-B (#174597), and lentiviral packaging vector plasmids, pCMV-dR8.2 dvpr (#8455) and pCMV-VSV-G (#8454), were obtained from Addgene (Watertown, MA, USA). The lentiviral vector (pLVX-IRES-Puro plasmid, Clontech Laboratories, Inc., Mountain View, CA, USA) was utilized to construct control, *Gmcsf,* and *Ifnb1* expression vectors. Lentiviruses for control vector, *Gmcsf*, or *Ifnb1* expression were produced in 293FT cells using a co-transfection approach. Briefly, plasmid mixtures, including either the control vector, the *Gmcsf*- or *Ifnb1*-expressing plasmid, alongside pCMV-dR8.2 dvp and pCMV-VSV-G, were co-transfected into 293FT cells at ~80% confluence using Lipofectamine 2000 (#11668019, Thermo Fisher Scientific), according to the manufacturer’s instructions. Supernatants containing lentiviral particles were harvested 48–72 h post-transfection and concentrated for use in transductions. To generate control, GM-CSF-expressing, or IFNB1-expressing MOC2 cell lines, MOC2 cells were plated 24 h before transduction. When the cells reached ~50% confluence, lentiviral transduction was performed using a multiplicity of infection (MOI) of 5 in an Opti-MEM™ medium (#31985062, Thermo Fisher Scientific) supplemented with 10 µg/mL polybrene (#TR-1003-G; Sigma–Aldrich, St. Louis, MO, USA) at 37 °C for 5 h. Following transduction, cells were cultured in complete IMDM/F12 media for two days. Successfully transduced cells were selected by treating them with puromycin (2.5 µg/mL, #A11138-03, Thermo Fisher Scientific) for four days.

### 2.3. Cell Viability and Proliferation Assay

Cell viability was assessed using Cell Counting Kit-8 (CCK-8; #K1018, APExBIO, Houston, TX, USA) following the manufacturer’s instructions. Briefly, cells were seeded in a 96-well plate at a density of 3000 cells/well. After incubation, CCK-8 solution (10 µL) was added to each well containing 100 µL of culture medium and further incubated for ~2 h at 37 °C. The absorbance was measured at 450 nm using a microplate reader.

### 2.4. Bone Marrow Cell Derived Dendric Cells (BMDCs) Induction

Bone marrow cells were isolated from the femurs and tibias of 5–6-week-old C57BL/6 mice (#000664, the Jackson Laboratory, Farmington, CT, USA). The cells were plated at a density of 1 × 10⁶ cells per well in a 6-well culture plate in a bone marrow cell culture medium. After 48~72 h of incubation, the medium was replaced with fresh medium or a 1:1 mixture of fresh and conditioned media from MOC2 cancer cells, supplemented with 5 ng/mL GM-CSF. The cells were cultured for 7 days to promote differentiation into dendritic cells (DCs).

### 2.5. Flow Cytometric Analysis

For apoptosis analysis, 3 × 10^5^ cells were seeded per well in a 6-well plate. After incubation for two days, the cells were collected and stained with FITC-conjugated Annexin V and propidium iodide (PI) using the FITC Annexin V Apoptosis Detection Kit (#556547, BD Biosciences, CA, USA) following the manufacturer’s protocol. Stained cells were incubated in the dark for 15 min before flow cytometry. Annexin V-positive/PI-negative (early apoptosis) and Annexin V-positive/PI-positive (late apoptosis) populations were identified as apoptotic cells, and their percentages were analyzed. Flow cytometric experiments were performed using a flow Cytometer (#A00-1-1102, Beckman Coulter, Brea, CA, USA) equipped with CytExpert (Version 2.4) software, and the data were analyzed using FlowJo10 (FlowJo, Ashland, OR, USA).

For cell surface antigen expression or antigen presentation, the following antibodies were used: FITC anti-mouse CD8α antibody (#155004), APC anti-mouse CD11c antibody (#117310), PE anti-mouse CD11c antibody (#117307), FITC anti-mouse/human CD11b antibody (#101206), APC anti-mouse H-2K^b^ antibody (#116517), APC anti-mouse H-2K^b^ bound to SIINFEKL antibody (#141605), and FITC anti-mouse I-A/I-E antibody (#107605). All were purchased from BioLegend (San Diego, CA, USA). To exclude dead cells, 4′,6-diamidino-2-phenylindole (DAPI; #62248, Thermo Fisher Scientific) staining was used.

### 2.6. Enzyme-Linked Immunosorbent Assay (ELISA) for Cytokine Detection

Cytokine levels were measured using a Sandwich ELISA. Mouse GM-CSF ELISA Kit (#KE10015; Proteintech, Rosemont, IL, USA) and Mouse IFNB1 ELISA Kit (#439407; BioLegend) were used according to the manufacturers’ instructions. Briefly, 96-well plates pre-coated with capture antibodies were blocked and incubated with samples and standards. Detection antibodies and streptavidin–HRP were added sequentially, followed by a substrate for color development. Absorbance was measured at 450 nm, and cytokine concentrations were calculated using standard curves.

### 2.7. Nucleic Acid Extraction and PCR Assay

RNeasy Mini Kit (#74106, Qiagen, Germantown, MD, USA) was used for the isolation of total RNA from cultured cells following the manufacturer’s instructions. A NanoDrop 2000c spectrophotometer (ND-2000c, Thermo Scientific, Waltham, MA, USA) was employed to measure RNA concentrations. RNA samples were stored at −80 °C. Real-time quantitative reverse transcription PCR (qRT-PCR) was performed in a total volume of 20 µL containing 10 µL of 2× Luna Universal One-Step Reaction Mix (#E3005, New England Biolabs, Ipswich, MA, USA), 1 µL of RT Enzyme Mix (20×), 1 µL of 5 µM primer for each primer per reaction, 2 µL of the RNA dilution (100 ng/µl), and 6 µL of water. The primers used in this study are listed in Table 1. The PCR cycling on a StepOnePlus^TM^ Real-Time PCR System (Applied Biosystems, Life Technologies, CA, USA) was performed as follows: a reverse transcription step (55 °C, 15 min) and an initial denaturation step (95 °C, 1 min), followed by 45 cycles of denaturation (95 °C, 10 s) and extension (60 °C, 60 s) and a single cycle of melting curve measurement step (95 °C for 15 s, 60 °C for 15 s, and 95 °C for 15 s). The fold change in gene expression levels relative to the control, normalized to GAPDH mRNA, was calculated using the 2^−∆∆Ct^ method.

### 2.8. Western Blot

Protein extraction was performed using RIPA buffer (10 mM Tris, pH 7.4; 150 mM NaCl; 1% NP-40; 1% sodium deoxycholate; 0.1% SDS; protease inhibitors). Protein concentrations were determined using the Bradford protein assay. Equal amounts of protein (typically 20 µg) were loaded onto SDS-PAGE (sodium dodecyl sulfate–polyacrylamide gel electrophoresis) gels and transferred onto membranes. Cell lysates were separated by 10% SDS-PAGE and transferred onto 0.2 μm nitrocellulose membranes (#1704158, Bio-Rad Laboratories, Hercules, CA, USA). Membranes were incubated overnight at 4 °C with specific primary antibodies (1:1000 dilution). β-actin served as the loading control. The antibodies used in this study were sourced as follows: GM-CSF (#17762-1-AP) and cGAS (#29958-1-AP) were obtained from Proteintech. β-actin (#4967) and STING (#13647) were obtained from Cell Signaling Technology (Danvers, MA, USA). Immunoblotting was carried out using HRP-conjugated secondary antibodies (anti-rabbit IgG or anti-mouse IgG, 1:2000 dilution) for detection, and specific protein bands were visualized with an enhanced chemiluminescence (ECL) (#1705062, Bio-Rad) detection system. Protein band intensities were quantified using ImageJ software (https://imagej.net/ij/download.html (accessed on 19 February 2025), ImageJ, National Institutes of Health, Bethesda, MD, USA).

### 2.9. SIINFEKL Peptide Pulsing of Cells

To evaluate antigen presentation, cells were pulsed with the SIINFEKL peptide (OVA257–264; #17-5743-82, Thermo Fisher Scientific). Briefly, cells were plated at a density of 0.5 × 10^6^ cells per well in a 6-well plate and allowed to adhere overnight. The next day, the SIINFEKL peptide was diluted in sterile PBS to a final concentration of 100 μg/mL. Resuspended cells were incubated with the peptide for 1–2 h at 37 °C in a 5% CO_2_ incubator. After incubation, cells were washed three times with sterile PBS to remove unbound peptide and prepared for subsequent analysis using flow cytometry with an H-2K^b^ bound to the SIINFEKL antibody.

### 2.10. Tumor Models in Mice

Five- to six-week-old C57BL/6 (#000664) and 5–6-week-old NOD scid IL-2Rγ^-/-^ (NSG) (#005557) mice were purchased from the Jackson Laboratory. A total of 5 million mouse cancer cells, suspended in 100 μL of medium containing 50% Matrigel basement membrane matrix (#354234, BD Biosciences), were subcutaneously inoculated into the flanks of the mice. Tumor growth was monitored by measuring tumor sizes with a caliper every 2–3 days. At the experimental endpoints, mice were euthanized, and tumors were harvested for further analysis. A tumor dissociation experiment was performed using the mouse tumor dissociation kit purchased from the Myltenyi Biotec (#130-096-730, Auburn, CA, USA) according to the manufacturer’s instructions. All animal experiments were carried out in accordance with protocols approved by the University Animal Care and Use Committee and adhered to the guidelines outlined in the Guide for the Care and Use of Laboratory Animals by the National Institutes of Health.

### 2.11. Tissue Staining and Immunohistochemical Analysis

Tissues were fixed in 10% neutral buffered formalin solution overnight, embedded in paraffin, sectioned at 5 µm, and stained with hematoxylin and eosin (H&E) following standard protocols. Immunohistochemical (IHC) staining was performed as previously described [22,23]. Briefly, paraffin-embedded tissue sections were deparaffinized and rehydrated. To inhibit endogenous peroxidase activity, sections were incubated with 0.3% hydrogen peroxide, followed by a thorough rinse with PBS (pH 7.6). Sections were subjected to antigen retrieval using 0.05% citraconic anhydride as the retrieval solution at pH 7.4, 95 °C for 60 min. The slides were then incubated with primary antibodies (1:200 dilution) targeting cGAS (#29958-1-AP, Proteintech), IFNB1 (#27506-1-AP, Proteintech), or STING (#13647, Cell Signaling Technology) overnight at 4 °C. After five 5-min washes in PBS, the sections were incubated with secondary antibodies (HPR-conjugated goat anti-rabbit IgG and goat anti-mouse IgG, 1:200 dilution, Sigma) for 2 h at room temperature. Following an additional wash for 5 min, repeated 5 times, the tissues were incubated with DAB (#SK-4100, Vector Laboratories, Burlingame, CA, USA) for 1–5 min to visualize staining. Finally, the sections were counterstained with Gill’s Hematoxylin#1 (#GHS132, Sigma).

### 2.12. Statistics

All statistical analyses were performed using GraphPad Prism 8. The normality of the data was assessed using the Shapiro–Wilk test. Data are expressed as mean ± standard deviation (SD). For comparisons, statistical analyses were conducted using a two-tailed Student’s *t*-test for two-group comparisons. A one-way ANOVA was used for experiments involving more than two groups, while a two-way ANOVA was employed for experiments with multiple time-point measurements, followed by a post hoc test (e.g., Tukey’s test for pairwise comparisons). All tests were two-sided, and a *p*-value < 0.05 was considered statistically significant.

## 3. Results

### 3.1. Deficient Immune Cell Populations in MOC2 Tumors Compared to MOC1 Tumors in Immunocompetent Mice

**Figure 1:**
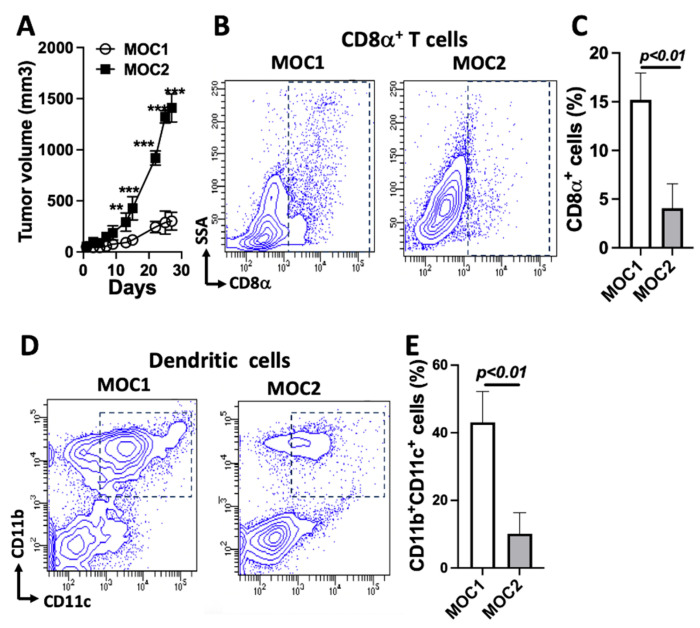
MOC1 and MOC2 tumors exhibit differences in growth and immune infiltration in C57BL/6 mice: (**A**) Tumor growth of MOC1 and MOC2 cells in C57BL/6 mice following subcutaneous inoculation of 5 × 10^6^ cells per mouse (*n* = 4). (**B**) Representative flow cytometry analysis showing a reduced number of CD8α^+^ cells in MOC2 tumors compared to MOC1 tumors. (**C**) Quantitative analysis of CD8α^+^ cells in MOC1 and MOC2 tumors (*n* = 3). (**D**) Representative flow cytometry analysis showing a reduced number of CD11b^+^CD11c^+^ cells, representing dendritic cells, in MOC2 tumors compared to MOC1 tumors. (**E**) Quantitative analysis of CD11b^+^CD11c^+^ cells in MOC1 and MOC2 tumors (*n* = 3). *p*-values are shown. ** *p* < 0.01; *** *p* < 0.001.

It has been reported that MOC1 cells exhibit an indolent growth phenotype, while MOC2 cells exhibit aggressive growth [15]. We established mouse MOC1 and MOC2 tumors in C57BL/6 mice. We evaluated tumor growth in mouse models following the subcutaneous inoculation of equal numbers of MOC1 and MOC2 cells (Figure 1A) and found that MOC2 tumors exhibited significantly faster progression compared to MOC1 tumors, consistent with the discovery from previous research [16]. Tumor infiltration of T cells is critical for antitumor immunity [24]. Previous work has demonstrated that MOC1 tumors present a T-cell inflamed status, while MOC2 tumors exhibit a non-T-cell inflamed status [25,26]. Indeed, in our experiment, CD8α was used as a marker for cytotoxic T lymphocytes. A comparative analysis between the two tumor types revealed that MOC2 tumors contained significantly fewer CD8α^+^ T cells compared to MOC1 tumors, as determined using flow cytometry (Figure 1B,C). The recruitment of antigen-presenting cells, particularly dendritic cells, into the tumors is a critical factor that influences tumor development during tumorigenesis. There is strong evidence that the suppression of tumor-infiltrating DCs from the TME impairs their ability to initiate robust antitumor immunity and potentially contributes to tumor progression. DCs play a pivotal role in regulating the balance between CD8^+^ T-cell immunity and tolerance to tumor antigens [27,28]. A recent elegant study demonstrated that IFN-I activates MHC class I-dressed CD11b^+^ conventional DCs, promoting protective antitumor CD8^+^ T-cell immunity [29]. Utilizing CD11b and CD11c as surface markers to assess the amount of the tumor-infiltrating DCs, we found that MOC2 contained significantly fewer CD11b^+^CD11c^+^ cells compared to MOC1 tumors (Figure 1D,E). These data clearly demonstrate that the more progressive MOC2 tumors compared to MOC1 tumors in C57BL/6 mice were associated with a much lower infiltration of CD8α ^+^ T cells and CD11b^+^CD11c^+^ DCs.

### 3.2. Reduced cGAS-STING-IFN-I Signaling in MOC2 Tumors Compared to MOC1 Tumors

**Figure 2:**
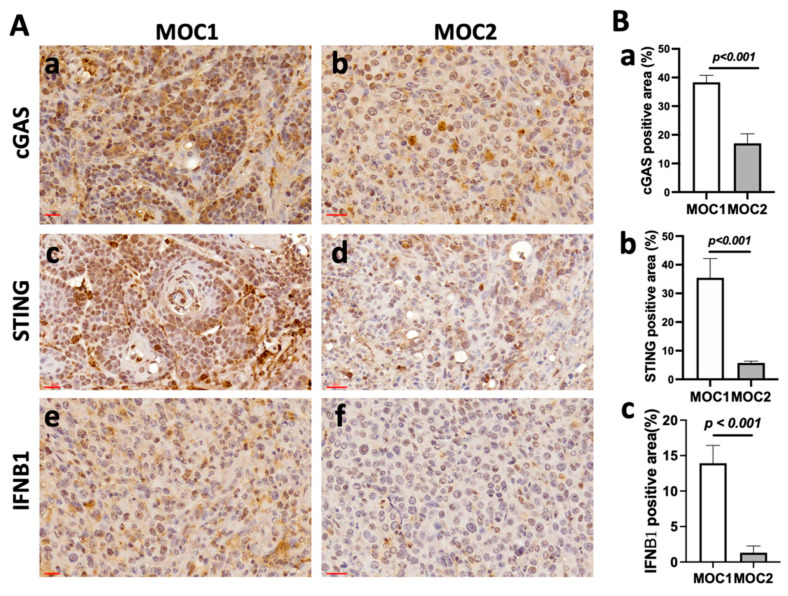
Differential expression of key regulator proteins in the cGAS-STING-IFN-I signaling pathway in MOC1 and MOC2 tumors in C57BL/6 mice: (**A**) Representative immunohistochemistry (IHC) analysis showing the expression of cGAS (**Aa**,**Ab**), STING (**Ac**,**Ad**), and IFNB1 (**Ae**,**Af**) in MOC1 and MOC2 tumors. Scale bar = 30 μm (insets). (**B**) Quantitative analysis of positive staining area for cGAS (**Ba**), STING (**Bb**), or IFNB1 (**Bc**) in MOC1 and MOC2 tumors. Data represent *n* = 3 tumors, with two slide replicates per tumor; *p*-values are shown.

The cytosolic DNA sensing pathway, which detects abnormal cytosolic DNA, has been recognized as a critical component of the innate immune system, playing a key role in defending against cancer. In many tumor cells, aberrant DNA accumulation stimulates cGAS-STING signaling, leading to the upregulation of IFN-I and proinflammatory cytokines. Upon activation by abnormal DNA, this pathway triggers specific immune responses that can influence various stages of tumorigenesis, including the initial transformation of malignant cells and the progression to metastasis [30]. To explore whether the cGAS-STING-IFN-I pathway contributes to the observed differential progression of MOC1 and MOC2 tumors, we compared the expression of the key regulators in this pathway, as well as cGAS, STING, and IFNB1 expression in MOC1 and MOC2 using an IHC analysis of the dissected tumors (H&E staining of the dissected tumors is shown in Supplementary Figure S1). As shown in Figure 2, IHC experiments revealed a consistent pattern of significantly fewer cGAS-positive (Figure 2Aa,b,Ba), STING-positive (Figure 2Ac,d,Bb), and IFNB1-positive cells (Figure 2Ae,f,Bc) in MOC2 tumors compared to MOC1 tumors. We also compared the expression of cGAS and STING in cultured MOC1 and MOC2 cells. The expression levels of cGAS and STING were significantly lower in MOC2 cells compared to MOC1 cells, suggesting that the observed differences in protein levels originate from the cancer cells rather than the stromal cells (Supplementary Figure S2). It has been reported that the downregulation of IFN-I signaling can reduce oncogene-induced senescence and promote tumorigenesis in chemically induced tumor models [31,32,33]. These data indicate that immune-cold MOC2 tumors may have more suppressed innate immune cGAS-STING-IFN-I signaling than MOC1 tumors, contributing to the more aggressive progression of MOC2 tumors.

### 3.3. MOC2 Cells Inhibit Bone-Marrow-Cell-Derived DC Differentiation

**Figure 3:**
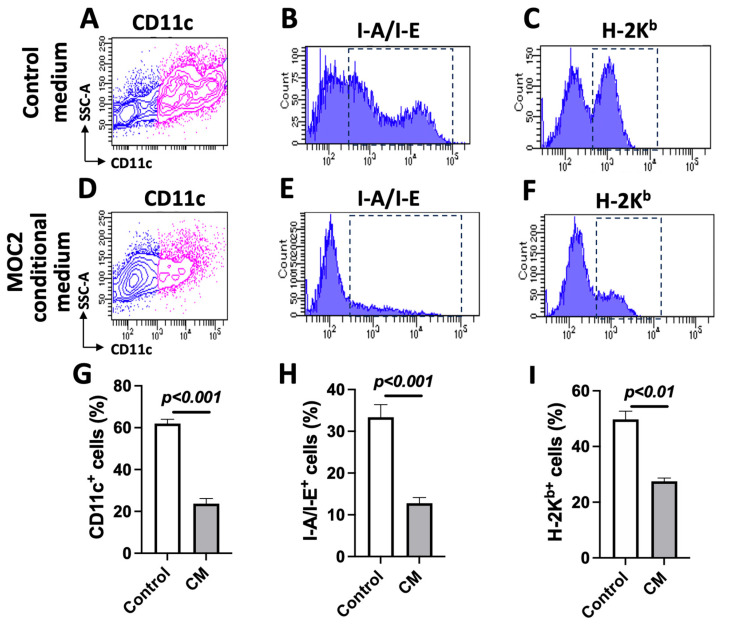
MOC2-conditioned medium reduces mouse bone marrow-derived dendritic cell differentiation and marker expression: (**A**–**F**) Representative flow cytometry analysis of marker expression in mouse bone marrow cell-derived dendritic cells. CD11c expression (**A**,**D**), I-A/I-E expression (**B**,**E**), and H-2Kᵇ expression (**C**,**F**) were analyzed in the cells grown in complete RPMI-1640 medium (control) (**A**–**C**) or 50% MOC2-conditioned medium (**D**–**F**) supplemented with 5 ng/mL GM-CSF. (**G**–**I**) Quantitative analysis of CD11c positive cells (**G**), I-A/I-E positive cells (**H**), and H-2K^b^ positive cells (**I**). CM, conditioned medium. Data represent *n* = 3; *p*-values are indicated.

The significant deficiency of DCs in MOC2 tumors compared to MOC1 tumors prompted us to assess how MOC2 cells influence the differentiation and functional status of DCs. DCs are regarded as the most effective professional antigen-presenting cells. They play a crucial role in initiating adaptive immune responses by capturing, processing, and presenting antigens, including tumor antigens. DCs present these antigens to naive CD8^+^ T cells via MHC class I proteins and to CD4^+^ T cells via MHC class II proteins [34]. The MHC-mediated presentation of tumor antigens is essential for T-cell-driven immune responses. Specifically, HLA class I molecules present antigens to T-cell receptors (TCRs) on CD8^+^ T cells, while HLA class II molecules, predominantly expressed on antigen-presenting cells, present antigens to TCRs on CD4^+^ T cells. The absence of HLA expression can impair T-cell engagement through their TCRs and hinder their proliferation, potentially leading to decreased T-cell infiltration within the TME. Additionally, MHC-I downregulation has been observed in various cancers and is often associated with a poorer prognosis [35]. In mice, H-2K^b^ serves as the equivalent of human HLA class I, while I-A and I-E correspond to MHC class II. We performed flow cytometry analysis on mouse bone marrow cells after they were cultured in complete RPMI1640 medium or 50% MOC2-conditioned medium in the presence of 5 ng/mL GM-CSF for 7 days. The results showed that, compared to the control medium, MOC2-conditioned media significantly reduced the percentage of BMDCs that were CD11c-positive cells (a marker commonly used for DCs and often associated with differentiation) (Figure 3A vs. Figure 3D, and Figure 3G), I-A/I-E-positive cells (antigen presentation machinery proteins) (Figure 3B vs. Figure 3E, and Figure 3H), and H-2K^b^-positive cells (Figure 3C vs. Figure 3F, and Figure 3I). Importantly, the inhibition was specific to the MOC2-conditioned medium, as DC differentiation was not impaired in the MOC1-conditioned medium compared to the control medium (Supplementary Figure S3). These findings suggest that MOC2 cells impaired DC differentiation and antigen presentation capability, underscoring the critical role of the cancer cell-mediated suppression of DC responses in promoting immune evasion.

### 3.4. MOC2 Cells Retain the Ability to Present Antigen in Response to Transfected DNA or IFNB1 Expression

**Figure 4:**
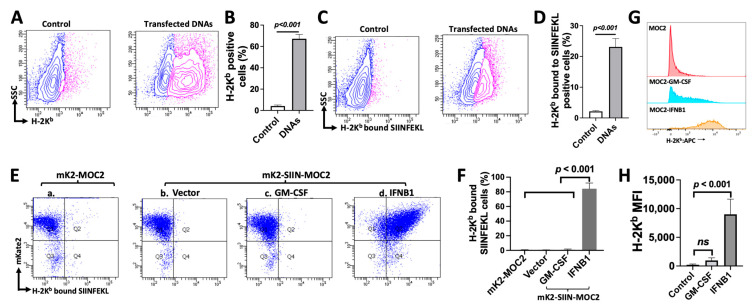
Effects of transfected plasmid DNAs or expressed IFNB1 on H-2K^b^ and H-2K^b^-bound SIINFEKL expression: (**A**) Transfection of plasmid DNAs (right panel), leading to increased H-2K^b^ expression in MOC2 cells compared to control MOC2 cells (left panel). (**B**) Quantification of H-2K^b^ positive cells in control and plasmid DNA transfected MOC2 cells (*n* = 4). (**C**) Transfected plasmid DNA (right panel) increases H-2K^b^-bound SIINFEKL in MOC2 cells compared to control cells (left panel) after pulsing with the SIINFEKL peptide. (**D**) Quantification of the increase in H-2K^b^-bound SIINFEKL-positive cells in the plasmid DNA transfected cells compared to control cells (*n* = 4). (**G**) Viral-mediated transduction of IFNB1 (illustrated in orange, bottom panel) but not GM-CSF (blue, middle panel) increases H-2K^b^ expression compared to the control (Pink, top panel) MOC2 cells. (**H**) Mean fluorescence intensity (MFI) of H-2K^b^ expression in control, GM-CSF-expressing MOC2 cells, and IFNB1-expressing MOC2 cells (*n* = 3). (**E**) Expression of IFNB1 (**Ed**) in mK2-SIIN-MOC2 cells, which express the fluorescent protein mKate2 and SIINFEKL, significantly increases H-2K^b^-bound SIINFEKL presentation compared to either the vector control (**Eb**) or the GM-CSF-expressing (**Ec**) cells. mK2-MOC2 cells (**Ea**) were used to indicate mKate2 expression as a control. mK2: mKate2; SIIN: SIINFEKL. (**F**) Quantification of H-2K^b^-bound SIINFEKL positive cells in mk2-MOC2, vector control-expressing, GM-CSF-expressing, IFNB1-expressing-mK2-SIIN-MOC2 cells (*n* = 3). *p*-values are indicated.

While MOC1 tumors respond to ICB and STING agonists, suppressing tumor growth, MOC2 tumors are resistant to these treatments [18]. In our study, compared to MOC1 tumors, MOC2 tumors exhibited suppression in the cGAS-STING-IFN-I signaling pathway, suggesting dysfunctional DNA sensing. To assess whether MOC2 cells respond to abnormally increased cytoplasmic DNA in vitro, plasmid DNA was transfected into MOC2 cells. Compared to control MOC2 cells, which were treated with the transfection reagent without plasmid, plasmid transfection significantly increased the expression of H-2K^b^, a mouse MHC-I protein involved in antigen presentation, on the surface of plasmid DNA-transfected MOC2 cells (Figure 4A,B). To evaluate whether the enhanced membrane expression of H-2K^b^ was functional, we treated plasmid DNA-transfected MOC2 cells with SIINFEKL peptide pulsing and assessed antigen presentation by measuring H-2K^b^-bound SIINFEKL using flow cytometry. This analysis revealed a significant increase in H-2K^b^-bound SIINFEKL on MOC2 cells after SIINFEKL peptide pulsing (Figure 4C,D). These results indicate that although antigen presentation was suppressed in MOC2 cells without stimulation, the activation of the DNA-sensing pathway may restore antigen presentation in MOC2 cells.

**Figure 5:**
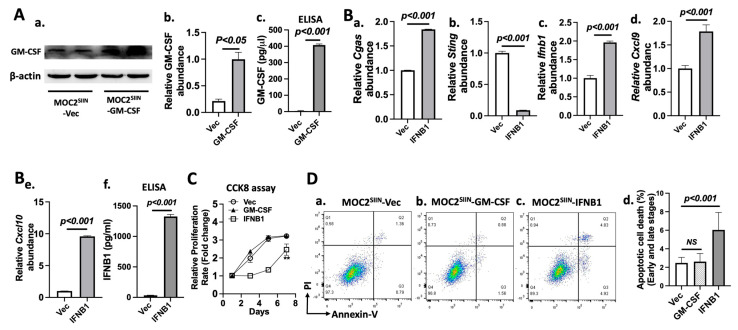
Effects of IFNB1 or GM-CSF expression in MOC2^SIIN^ cells: (**A**) (**Aa**) GM-CSF protein levels in MOC2^SIIN^ cells expressing either vector control or exogenous GM-CSF, as determined using Western blot analysis. (**Ab**) Quantification of GM-CSF protein levels normalized to β-actin (*n* = 2). (**Ac**) GM-CSF concentration in the cell culture supernatant of MOC2^SIIN^ cells expressing either vector control or exogenous GM-CSF, as measured using ELISA (*n* = 3). (**B**) Relative mRNA expression of *Cgas* (**Ba**), *Sting* (**Bb**), *Ifnb1* (**Bc**), *Cxcl9* (**Bd**), or *Cxcl10* (**Be**) in MOC2^SIIN^ cells expressing either vector control or exogenous IFNB1, as determined using RT-PCR analysis (*n* = 4). (**Bf**) IFNB1 concentration in the cell culture supernatant of MOC2 expressing either vector control or IFNB1, as measured using ELISA (*n* = 3). (**C**) Cell growth curves of MOC2^SIIN^ cells transduced with vector control, GM-CSF, or IFNB1, as measured using the CCK-8 assay (*n* = 6). (**D**) Flow cytometric analysis of apoptotic cell death in vector control (**Da**), GM-CSF-transduced (**Db**), and IFNB1-transduced (**Dc**) MOC2^SIIN^ cells. (**Dd**) Quantification of apoptotic cell death in both early and late stages in vector control, GM-CSF-transduced, and IFNB1-transduced MOC2^SIIN^ cells (*n* = 6). Abbreviation: Vec: vector-expressing MOC2^SIIN^, GM-CSF: GM-CSF-expressing MOC2^SIIN^, IFNB1: IFNB1-expressing MOC2^SIIN^. ** *p* < 0.01. *p*-values are shown. The original blots can be found in Supplementary File S1.

IFNB1 is known for its role in stimulating antiviral responses and upregulating MHC-I expression. To evaluate whether IFNB1 signaling directly stimulates antigen presentation in MOC2 cells, we set out to investigate its modulation of the antigen presentation machinery. GM-CSF is primarily known for its role in the proliferation, differentiation, and activation of myeloid cells, such as granulocytes and macrophages [36]. While GM-CSF can promote the maturation of DCs and enhance their antigen-presenting capabilities in certain contexts, its direct impact on MHC-I expression and peptide presentation by tumor cells, such as MOC2, remains unclear. We used GM-CSF as a control in this study and expressed either GM-CSF or IFNB1 via viral transduction. The experiments were carried out in two established MOC2 sublines, MOC2^Ctl^ (expressing mKate2 but not SIINFEKL) and MOC2^SIIN^ (expressing both mKate2 and SIINFEKL). Using MOC2^Ctl^ cells as a control for fluorescent mKate2 expression (Figure 4Ea), we transduced MOC2^SIIN^ cells to express either GM-CSF or IFNB1. The successful expression of GM-CSF was confirmed using Western blotting and ELISA. Compared to the control vector group, GM-CSF viral transduction showed a significant increase in both protein expression (Figure 5Aa,b) and GM-CSF secretion in the cell culture medium (Figure 5Ac), respectively. The increase in *Ifnb1* expression in IFNB1-transduced MOC2^SIIN^ cells was confirmed using RT-PCR (Figure 5Bc) and by detecting secreted IFNB1 in the cell culture supernatant using ELISA (Figure 5Bf). These data indicate that the ectopic expression of either IFNB1 or GM-CSF in MOC2^SIIN^ cells resulted in a significant upregulation of their respective protein products and secretion. Importantly, we found that more than 80% of MOC2^SIIN^ cells exhibited a significant increase in H-2K^b^-bound SIINFEKL expression following viral-mediated IFNB1 transduction (Figure 4Ed,F). In contrast, either viral-mediated GM-CSF transduction (Figure 4Ec,F) or control vector (Figure 4Eb,F) did not lead to any significant increase in H-2K^b^-bound SIINFEKL expression. To further examine the impact of IFNB1 or GM-CSF on antigen presentation machinery in cancer cells, we analyzed the surface expression of H-2K^b^ on MOC2 cells expressing either IFNB1 or GM-CSF via viral transduction. Using flow cytometry, we found that MOC2 cells expressing IFNB1, but not those expressing GM-CSF, exhibited significantly enhanced H-2K^b^ expression on their cell membrane (Figure 4G,H).

Taken together, the lack of effect of GM-CSF on either H-2K^b^ expression or H-2K^b^-bound SIINFEKL expression suggests that GM-CSF did not directly influence antigen processing or presentation mechanisms in MOC2 cells. In contrast, the increase in H-2K^b^ and H-2K^b^-bound SIINFEKL expression following plasmid DNA transfection or IFNB1 transduction indicates that these interventions directly enhanced MHC-I antigen presentation mechanisms in the immune “cold” MOC2 cells. These findings suggest that, despite being ICB-resistant and/or deficient in the DNA sensing pathway compared to MOC1, MOC2 cells can still be stimulated and retain the capacity for MHC-I-restricted antigen presentation through either DNA sensing or direct INFB1 expression.

### 3.5. Effects of IFNB1 Expression on Gene Expression, Cell Proliferation, and Apoptotic Cell Death in MOC2 Subline Cells

Since the viral transduction-mediated expression of IFNB1 but not GM-CSF affected the antigen presentation machinery of MOC2 subline cells, we evaluated how IFNB1 expression impacts immune-modulating genes in these cells. We assessed the expression of key genes in the cGAS-IFN-I signaling pathway, as well as pathway-activated genes in MOC2^SIIN^ cells transduced with *Ifnb1* in vitro. The IFN-I signals through its receptor [10] to activate IFN-stimulated genes (ISGs), including key chemokines like CXCL9 (C-X-C motif chemokine 9) and CXCL10. These chemokines are crucial for attracting CD8^+^ cytotoxic T cells to the tumor microenvironment [37]. RT-PCR analysis showed that the expression levels of genes in the cGAS-IFN-I pathway and its downstream targets, including *Cgas* (Figure 5Ba)*, Ifnb1* (Figure 5Bc)*, Cxcl9* (Figure 5Bd)*,* and *Cxcl10* (Figure 5Be)*,* were significantly increased by the ectopic *Ifnb1* expression compared to the vector control group. Interestingly, *Sting* expression was significantly decreased under the same conditions (Figure 5Bb). These findings suggest that enforced *Ifnb1* expression can upregulate both *Cgas* and *Ifnb1* itself; however, this did not necessarily lead to increased *Sting* expression. Instead, the downregulation of *Sting* may be attributed to a negative feedback mechanism triggered by the ectopic IFNB1 expression, preventing the overactivation of the pathway.

It has been reported that IFNB1 could either suppress [38] or promote [39] tumor growth. To evaluate the effects of viral-mediated expression of IFNB1 or GM-CSF on MOC2^SIIN^ cell proliferation, we performed a CCK8 assay to compare proliferation rates over a 7-day period. The growth curves (Figure 5C) revealed that MOC2^SIIN^ cells expressing IFNB1 exhibited a significantly lower proliferation rate compared to the other sublines up to day 5 of the experimental period, while MOC2^SIIN^ cells with GM-CSF expression showed no detectable change in proliferation compared to control MOC2^SIIN^ cells. However, IFNB1-expressing MOC2^SIIN^ cells exhibited accelerated cell proliferation from day 5 to day 7 compared to controls (Figure 5C). These data indicate that viral-mediated IFNB1 expression, but not GM-CSF expression, significantly impacted MOC2^SIIN^ cell proliferation.

We next evaluated apoptotic cell death in these cells to determine whether IFNB1 expression would result in abnormal apoptosis. Flow cytometry analysis revealed the apoptotic cells, including both the early and late stages, were significantly increased from 5.3% in the control cells (Figure 5Da,d) to 9.1% in the MOC2^SIIN^ cells expressing IFNB1 (Figure 5Dc,d), indicating that IFNB1 overexpression could result in more apoptotic cell death. On the contrary, the proportion of apoptotic cells was not significantly changed in the cells expressing GM-CSF compared to the control cells (Figure 5Db,d). Therefore, the ectopic expression of IFNB1, but not GM-CSF, promoted apoptotic cell death in these cells.

These data suggest that ectopic expression of IFNB1, but not GM-CSF, in the MOC2 subline cells altered cell proliferation and promoted apoptosis.

### 3.6. IFN-I Induces Adaptive Antitumor Immunity-Dependent Tumor Regression, Which Is Enhanced Using GM-CSF

**Figure 6:**
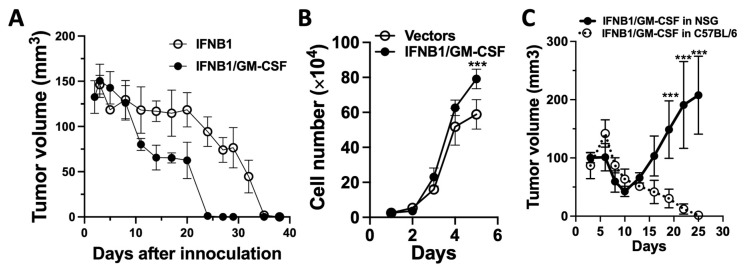
GM-CSF expression promotes IFNB1-mediated regression of MOC2^SIIN^ tumors via antitumor immunity: (**A**) Earlier regression of mixed MOC2^SIIN-IFNB1^/MOC2^SIIN-GM-CSF^ tumors (*n* = 4), which express both IFNB1 and GM-CSF, in C57BL/6 mice after subcutaneous inoculation of 5 × 10^6^ cells (50% IFNB1-expressing and 50% GM-CSF-expressing MOC2^SIIN^ cells), compared to MOC2^SIIN-IFNB1^ tumors expressing IFNB1 alone (50% IFNB1-expressing MOC2^SIIN^ cells and 50% MOC2^SIIN^ cells) (*n* = 3). (**B**) Cell growth curves over 5 days of heterogeneous MOC2^SIIN^ cells (10% IFNB1-expressing, 10% GM-CSF-expressing MOC2^SIIN^, and 80% MOC2^SIIN^) and control MOC2^SIIN^ cells (20% MOC2^SIIN^ cells transduced with vectors and 80% MOC2^SIIN^) in vitro (*n* = 3). (**C**) Regression of heterogeneous tumors (comprising 10% IFNB1-expressing, 10% GM-CSF-expressing MOC2^SIIN^, and 80% MOC2^SIIN^) in C57BL/6 mice following subcutaneous inoculation of 5 × 10^6^ cells per mouse compared to the same heterogeneous tumors in NSG mice (*n* = 4). *** *p* < 0.001.

Since we observed that MOC2 tumors were deficient in CD8^+^ T cells and DCs compared to MOC1 tumors, we evaluated whether IFNB1 expression could induce adaptive antitumor immunity for MOC2 tumor control and whether GM-CSF, a cytokine that stimulates DC functions, could enhance IFNB1-induced tumor control. Our in vitro data demonstrated that transduction-mediated expression of IFNB1, but not GM-CSF, led to a modest yet significant increase in apoptotic cell death in MOC2^SIIN^ cells. While GM-CSF is known to promote the differentiation and maturation of DCs, which are key players in immune responses, IFN-I is selectively required by DCs for the immune rejection of tumors [40] and is essential for natural cancer immunosurveillance [10]. To evaluate the contribution of IFNB1 and GM-CSF expression by MOC2^SIIN^ to overall tumor control in vivo, we combined two cell populations: IFNB1-expressing MOC2^SIIN^ cells (MOC2^SIIN-IFNB1^) and GM-CSF-expressing MOC2^SIIN^ cells (MOC2^SIIN-GM-CSF^). A mixed cell population consisting of 50% MOC2^SIIN-IFNB1^ and 50% MOC2^SIIN-GM-CSF^ was compared to a population consisting of 50% MOC2^SIIN-IFNB1^ and 50% vector-transduced MOC2^SIIN^ cells (MOC2^SIIN-Vec^). A total of 5 × 10^6^ cells per mouse were inoculated subcutaneously into C57BL/6 mice, and tumor growth was monitored. Tumors in both groups developed similarly within the first 7 days, reaching a mean volume of approximately 135 mm^3^, followed by gradual tumor regression. However, the subsequent progression of the tumors differed significantly between the two groups. As shown in Figure 6A, the mixed population co-expressing GM-CSF and IFNB1 exhibited significantly faster tumor regression compared to the population with only IFNB1 expression. Notably, tumors derived from the GM-CSF and IFNB1 co-expression population became undetectable by day 24 and remained absent until the experiment’s endpoint, whereas three out of four tumors derived from the population with only IFNB1 expression became undetectable by day 35 and remained so thereafter. These observations suggest that while IFNB1 expression alone could induce MOC2^SIIN^ tumor regression, the co-expression of GM-CSF, likely by attracting DCs, was important for promoting MOC2^SIIN^ tumor control.

To directly confirm that adaptive immunity is essential for effective tumor control, we created another heterogeneous mixture of cancer cells expressing IFNB1 or GM-CSF. To simulate a heterogeneous tumor scenario, we generated a mixed cell population comprising 80% MOC2^SIIN^ cells, 10% MOC2^SIIN-IFNB1^ cells, and 10% MOC2^SIIN-GM-CSF^ cells (designated as the IFNB1/GM-CSF group) and compared it with a vector control group consisting of 80% MOC2^SIIN^ and 20% vector-transduced MOC2^SIIN^ cells. We first assessed the impact of co-expressing IFNB1 and GM-CSF by distinct cell populations on overall cell growth in vitro. As shown in the five-day cell growth curve of the two groups in Figure 6B, both groups were seeded at a density of 50,000 cells per well in 12-well plates on day 0. From day 1 to day 4, the growth rates of both groups were comparable. By day 5, the IFNB1/GM-CSF group exhibited a slightly higher proliferation rate than the vector control group. Thus, the transduced IFNB1 and GM-CSF in these heterogeneous cells did not slow down overall cell proliferation. We next examined whether the observed tumor regression was dependent on adaptive immunity by inoculating the heterogeneous IFNB1/GM-CSF cell mixture into immunocompetent C57BL/6 mice and immunodeficient NSG mice. NSG mice are widely used due to their severely immunodeficient status, characterized by the lack of functional T cells, B cells, and natural killer (NK) cells. This unique immunodeficient background makes NSG mice ideal for evaluating tumor growth in the absence of adaptive immunity while retaining certain innate immune components, such as macrophages and dendritic cells. We inoculated the heterogeneous cell mixtures from the IFNB1/GM-CSF group (total of 5 × 10^6^ cells per mouse) into both immunodeficient NSG mice and immunocompetent C57BL/6 mice and compared tumor growth. Our findings revealed that while tumors in C57BL/6 mice continued to regress from day 6 throughout the 27-day experiment, tumors in NSG mice progressed after an initial period of suppressed growth (Figure 6C). This suggests that an intact adaptive immune response in C57BL/6 mice was essential for sustained tumor regression. These results confirm that adaptive immune mechanisms were required for the effective regression of tumors formed by the heterogeneous IFNB1-expressing and GM-CSF-expressing MOC2^SIIN^ cells. Our data indicate that IFNB1, together with GM-CSF, may attract DCs to stimulate adaptive immunity, leading to MOC2^SIIN^ tumor regression.

### 3.7. IFN-I Signaling Engages Dendritic Cells to Induce Antitumor Immunity and Control Tumor Growth

**Figure 7:**
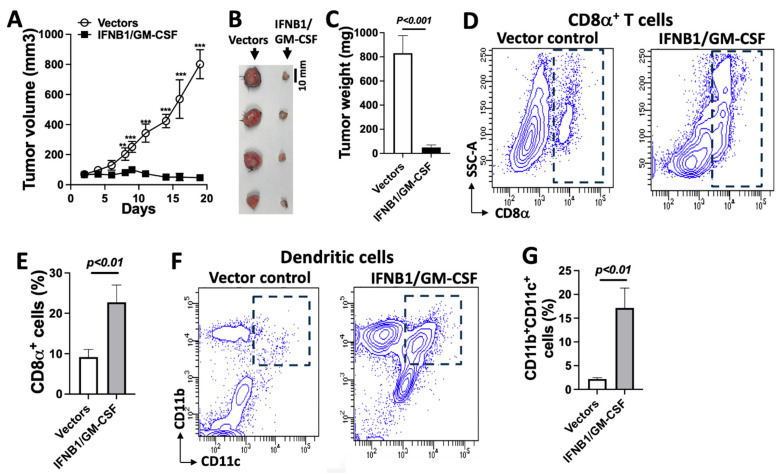
Co-expression of IFNB1 and GM-CSF in MOC2 tumors effectively suppresses tumor growth in C57BL/6 mice with elevated immune infiltration: (**A**) Reduced tumor growth of heterogeneous GM-CSF-expressing and IFNB1-expressing MOC2 cells (10% IFNB1-expressing MOC2, 10% GM-CSF-expressing MOC2, and 80% parental MOC2) compared to heterogeneous control MOC2 cells (20% MOC2 cells transduced with vector control and 80% parental MOC2) in C57BL/6 mice (*n* = 4) after subcutaneous inoculation of 5 × 10^6^ cells per mouse. (**B**) Tumor size of heterogeneous control MOC2 tumors and heterogeneous GM-CSF/IFNB1-expressing MOC2 tumors in C57BL/6 mice (*n* = 4; scale bar = 10 mm) and (**C**) tumor weight of dissected heterogeneous GM-CSF/IFNB1-expressing MOC2 tumors compared to control MOC2 tumors in C57BL/6 mice after the subcutaneous inoculation of 5 × 10^6^ cells per mouse (*n* = 4). (**D**) Representative flow cytometry analysis of the infiltration of CD8α^+^ T lymphocytes in dissected MOC2 tumors. (**E**) Quantitative analysis of infiltrated CD8α^+^ T lymphocytes in dissected MOC2 tumors (*n* = 3). (**F**) Representative flow cytometry analysis of infiltrated CD11b^+^CD11c^+^ dendritic cells in the dissected tumors. (**G**) Quantitative analysis of infiltrated CD11b^+^CD11c^+^ cells in the dissected MOC2 tumors (*n* = 3). ** *p* < 0.01; *** *p* < 0.001. *p*-values are shown.

To evaluate whether the co-expression of IFNB1 and GM-CSF can control tumor growth independently of exogenous antigens such as SIINFEKL in MOC2^SIIN^, we assessed their effects in MOC2 tumors lacking both SIINFEKL and fluorescent protein expression. A mixed cell population was created consisting of 80% parental MOC2 cells, 10% IFNB1-expressing MOC2 cells, and 10% GM-CSF-expressing MOC2 cells to simulate clinically relevant heterogeneous tumors. This mixture was inoculated into C57BL/6 mice at a total of 5 × 10⁶ cells per mouse. Compared to tumors derived from control MOC2 cells (80% parental MOC2 and 20% vector-transduced MOC2), tumors co-expressing IFNB1 and GM-CSF exhibited significantly inhibited growth (Figure 7A), reduced tumor size (Figure 7B), and decreased tumor weight (Figure 7C). Therefore, although this heterogeneous cancer cell population lacked both model antigen SIINFEKL and fluorescent protein expression, its in vivo tumor growth in immunocompetent mice was markedly suppressed. Our findings support the notion that IFNB1 may engage antitumor immunity through DCs to effectively control MOC2 tumor growth without requiring an exogenous antigen expression.

To further investigate whether the observed tumor suppression was mediated by enhanced antitumor immunity, we examined immune cell infiltration within the tumor microenvironment. Single-cell suspensions from the dissected tumors were analyzed using flow cytometry to quantify CD8α⁺ T cells and CD11b⁺ CD11c⁺ DCs. We found that tumors derived from the IFNB1/GM-CSF group exhibited a significant increase in both CD8α⁺ T cells (Figure 7D,E) and CD11b⁺ CD11c⁺ DCs (Figure 7F,G) compared to control tumors. These findings suggest that the co-expression of IFNB1 and GM-CSF in the heterogeneous MOC2 tumors enhanced the infiltration of immune effector cells, such as DCs and cytotoxic T lymphocytes, contributing to effective tumor growth suppression in C57BL/6 mice.

In summary, our data indicate that IFNB1 expression can restore dendritic cell infiltration and function, thereby activating adaptive antitumor immunity in immune-cold MOC2 tumors and leading to effective tumor control. This highlights the potential of targeting IFN-I signaling for treating aggressive HNSCC tumors.

## 4. Discussion

The most challenging issue in managing HNSCC with immunotherapy is the poor patient response, largely due to the ability of cancer cells to evade antitumor responses [7]. Utilizing well-established MOC1 and MOC2 mouse lines with known differential responses to ICB, we identified key mechanisms contributing to immune evasion by cancer cells. Our findings underscore the critical role of tumor-intrinsic IFN-I signaling in regulating immune cell function within the tumor microenvironment, particularly DCs and T cells, and shaping tumor immunogenicity, thereby enhancing antitumor immunity in HNSCC.

Advancing immunotherapies heavily rely on immunocompetent mouse models that replicate human disease [41]. Most HNSCCs of the oral cavity, oropharynx, and larynx are classified as cold tumors. In this study, we utilized the MOC1 and MOC2 OSCC cell lines as a model system to establish tumors in C57BL/6 mice. These cells are well characterized for their immune profiles, with MOC1 forming T-cell-inflamed tumors and MOC2 forming non-T-cell inflamed tumors [15,17,42]. This approach enabled us to examine and compare T-cell-inflamed and non-T-cell-inflamed tumor microenvironments. Indeed, our findings confirmed differential immune cell infiltration and tumor progression patterns between MOC1 and MOC2 tumors, with MOC2 tumors exhibiting fewer immune infiltrates and more aggressive growth. This suggests a close link between the level of T-cell infiltration and tumor behavior, including immunogenicity. A critical challenge in treating HNSCC is the poor response to immunotherapy [4,5], mainly due to the lack of antitumor T cells within the tumors. MOC2 tumors have been reported to be resistant to combined STING agonist and ICB treatment, in contrast to MOC1 tumors [18]. In our in vitro studies, we found that bone marrow cells conditioned by MOC2 cells exhibited impaired differentiation into DCs when induced by GM-CSF. Moreover, our analysis of immune infiltrates indicated a significant deficiency of DCs in MOC2 compared to MOC1 tumors, suggesting that these immune-cold tumors are ineffective in antigen cross-presentation, which is essential for T-cell activation. Our data indicate that the rapid progression of MOC2 tumors may impair the functions of DCs and CD8+ T cells, likely contributing to their reported resistance to STING agonist combined with ICB treatment.

The activation of the cGAS-IFN-I pathway has been actively pursued as a treatment strategy for various types of cancers [12,14,43,44]. However, the efficacy of STING-activating agents in clinical trials has been limited. A significant challenge with these agents is the pathway’s presence across various immune cell types, where non-cell-type-specific activation—such as chronic stimulation of this pathway [45] or activation in some suppressive immune cells [46]—can paradoxically promote tumorigenesis and tumor progression. Emerging evidence suggests that the DNA-sensing innate immunity plays critical roles in inducing cancer cell necroptosis [47], enhancing cancer cell immunogenicity [11], and facilitating effective tumor antigen cross-presentation to T cells, thereby initiating antitumor immunity [14,48,49]. In mouse models, inflamed MOC1 tumors respond to combined STING agonist and ICB treatment, resulting in protective immunity, while the immune-cold MOC2 tumors do not [18]. Our IHC analysis revealed that cGAS, STING, and IFNB1 were markedly suppressed in the TME of MOC2 tumors compared to MOC1 tumors, indicating that deficient DNA sensing and innate immune signaling may contribute to the immune evasion of immune-cold MOC2 tumors. The reduced expression of cGAS, STING, and IFN-I proteins in MOC2 tumors highlights the importance of this pathway in modulating tumor aggressiveness and immune responsiveness. In vitro, the impaired differentiation of bone marrow cells into DCs following exposure to MOC2-conditioned media indicates a crucial link between tumor-intrinsic innate immune signaling and the suppression of functional DC maturation. Interestingly, IFNB1 transduction in MOC2 cells upregulated the expression of *Cgas* and *Ifn-I* mRNA, whereas *Sting* expression was downregulated. Although it has been reported that stimulating STING may induce T-cell responses, our findings suggest that IFN-I-induced alterations in DC functions, as well as MHC-I-restricted antigen presentation such as H-2K^b^ and H-2K^b^-bound SIINFEKL expression, were associated with elevated IFN-I expression rather than STING in MOC2 cells. These results highlight the potential of harnessing tumor-intrinsic IFN-I signaling as an alternative approach to enhance cancer cell immunogenicity and antigen cross-presentation in MOC2 tumors.

There is growing evidence supporting the underutilized potential of the dendritic cell compartment in enhancing antitumor T-cell immunity. The intratumoral phase of the anti-cancer T-cell response, orchestrated by tumor-residing conventional type 1 DCs (cDC1), plays a pivotal role in determining whether immunity is protective or ineffective and has been leveraged for cancer therapy [50]. The crosstalk between cDC1s and T cells is critical for an effective ICB-mediated antitumor response, which can be augmented through DC-T-cell engagers [51]. In addition to antigen cross-presentation for priming antitumor CD8^+^ T-cell responses, MHC-I cross-dressing also contributes to this priming by DCs. Recent research has identified an activation state of CD11b^+^ DCs that can be triggered by tumor-derived IFN-I, enabling them to drive protective, systemic antitumor CD8^+^ T-cell immunity even in the absence of cDC1s capable of cross-presenting antigens [29]. Consistent with these findings, we observed that IFNB1 expression, further amplified by co-expression of GM-CSF, suppressed tumor growth in heterogenous MOC2 tumors in C57BL/6 mice. Specifically, we demonstrated that co-expression of exogenous IFN-I and GM-CSF in MOC2 tumors promoted the accumulation of CD11c^+^ CD11b^+^ DC and increased CD8^+^ T-cell infiltration, underscoring the therapeutic potential of targeting the IFN-I pathway in combination with DC recruitment to enhance antitumor immunity. Furthermore, the regression of tumors formed by IFNB1-expressing and GM-CSF-expressing MOC2 cell mixtures was shown to depend on adaptive immune responses. The observation that tumors regressed in C57BL/6 mice but not in NSG mice confirms that IFNB1 signaling induced adaptive immunity to suppress tumor growth. Additionally, we demonstrated that DNA transfection, or enforced IFN-I expression, but not GM-CSF expression, in MOC2 cells significantly upregulated antigen presentation on these cells, indicating that the deficient DNA sensing pathway profoundly reduced cancer cell immunogenicity to facilitate immune evasion. These findings suggest that targeting the defective DNA sensing pathway could provide at least two ways to overcome immune evasion: (1) stimulating DC function to enhance T-cell priming and (2) increasing antigen presentation on cancer cells to improve T-cell recognition and immune-mediated tumor clearance.

Our observations also raise the question of how IFNB1 signaling induces an antitumor immune response. In MOC2 tumors, IFNB1 expressions may primarily promote immune infiltration indirectly. At the early stage of tumor development, MOC2 tumors lack immune cells. In this context, IFNB1 released from IFNB1-expressing MOC2 cells can interact with IFN-I receptors (IFNAR1/2) on MOC2 cells, enhancing antigen presentation and inducing the expression of ISGs, including CXCL9 and CXCL10. These chemokines may recruit immune cells, particularly DCs and T cells, to the tumor microenvironment. When IFNB1 and GM-CSF are co-expressed in MOC2 tumors, GM-CSF can directly recruit immune cells [36], including DCs, further supporting antitumor immunity. Together, these interactions enhance dendritic cell-mediated antigen cross-presentation and T-cell activation, with CXCL9/10-driven chemotaxis facilitating immune cell infiltration into the tumor.

Our study provides insights into how DNA-sensing innate immunity influences tumor control, particularly in immune-resistant MOC2 tumors. Our findings suggest that inducing IFNB1 expression in MOC2 cells could enhance antitumor immunity in these resistant tumors. However, while oral cavity carcinoma is a major subtype of HNSCC, these cell lines may not fully capture the genetic, epigenetic, and immune heterogeneity across HNSCC subtypes [4]. Moreover, despite the advantages of mouse models in providing controlled experimental conditions [41], they do not fully replicate human tumor-immune interactions, including adaptive immune responses and the complexity of the tumor microenvironment. Further studies are needed to determine whether these findings extend to the broader spectrum of HNSCC. Our results highlight the potential of targeting tumor-intrinsic innate DNA-sensing pathways to enhance antitumor immunity, offering a promising approach to overcoming resistance in oral cancers that do not respond to current immunotherapies. However, translating these findings into clinical applications requires careful evaluation of interspecies differences, potential toxicity, and the complexities of human tumor-immune interactions. Future research should incorporate humanized mouse models and ex vivo experiments using patient-derived tissues to better bridge the gap between preclinical discoveries and clinical applications.

## 5. Conclusions

By leveraging murine MOC1 and MOC2 cell-derived tumors with differential dendritic cell and T-cell infiltration, our findings suggest that impaired DNA sensing through the cGAS-IFN-I signaling pathway in “immune-cold” HNSCC cells may contribute to antigen presentation dysfunction and DC deficiency, leading to immune evasion and therapeutic resistance to immunotherapy. IFNB1 expression, further amplified by GM-CSF co-expression within tumors, could significantly counteract immune evasion and lead to effective tumor control. Future mechanistic studies exploring the impact of DNA sensing by different cell types in a tumor via the cGAS-IFN-I pathway on DC and CD8+ T-cell functions, as well as on cancer cell immunogenicity, may lay the foundation for modulating this pathway to achieve effective cancer control.

## Figures and Tables

**Table 1:** Primers used for RT-PCR analysis.

Gene	Forward	Reverse
*Cgas*	GTG AGG ACC AAT CTA AGA CGA G	AGC ATG TTT TCT CTA TCC CGT G
*Sting*	GTC CTC TAT AAG TCC CTA AGC ATG	AAG ATC AAC CGC AAG TAC CC
*Ifnb*	CGA GCA GAG ATC TTC AGG AAC	TCA CTA CCA GTC CCA GAG TC
*Cxcl9*	AGT CCG CTG TTC TTT TCC TC	TGA GGT CTT TGA GGG ATT TGT AG
*Cxcl10*	TCA GCA CCA TGA ACC CAA G	CTA TGG CCC TCA TTC TCA CTG
*Gapdh*	TGC CCC CAT GTT TGT GAT GG	AAT GCC AAA GTT GTC ATG GAT GAC C

## Data Availability

All data will be made available upon request.

## Supplementary Materials

The following supporting information can be downloaded at: https://www.mdpi.com/article/10.3390/cancers17081279/s1, Figure S1: MOC1 and MOC2 tumors; Figure S2: Expression of cGAS and STING in MOC1 and MOC 2 cells; Figure S3: MOC1-conditioned medium does not reduce mouse bone marrow-derived dendritic cell differentiation; File S1: The original blots for Figure 5 and Figure S2.
