# Supplementary material for: Cancer Cell-Intrinsic Type I Interferon Signaling Promotes Antitumor Immunity in Head and Neck Squamous Cell Carcinoma
Source: Cancers (Basel). 2025 Apr 10;17(8):1279. doi: 10.3390/cancers17081279 (PMC12025670; doi:10.3390/cancers17081279)
Supplement: Supplementary file 1 [file cancers-17-01279-s001.zip › cancers-3515876-supplementary.pdf]

Supplementary Figure S1:

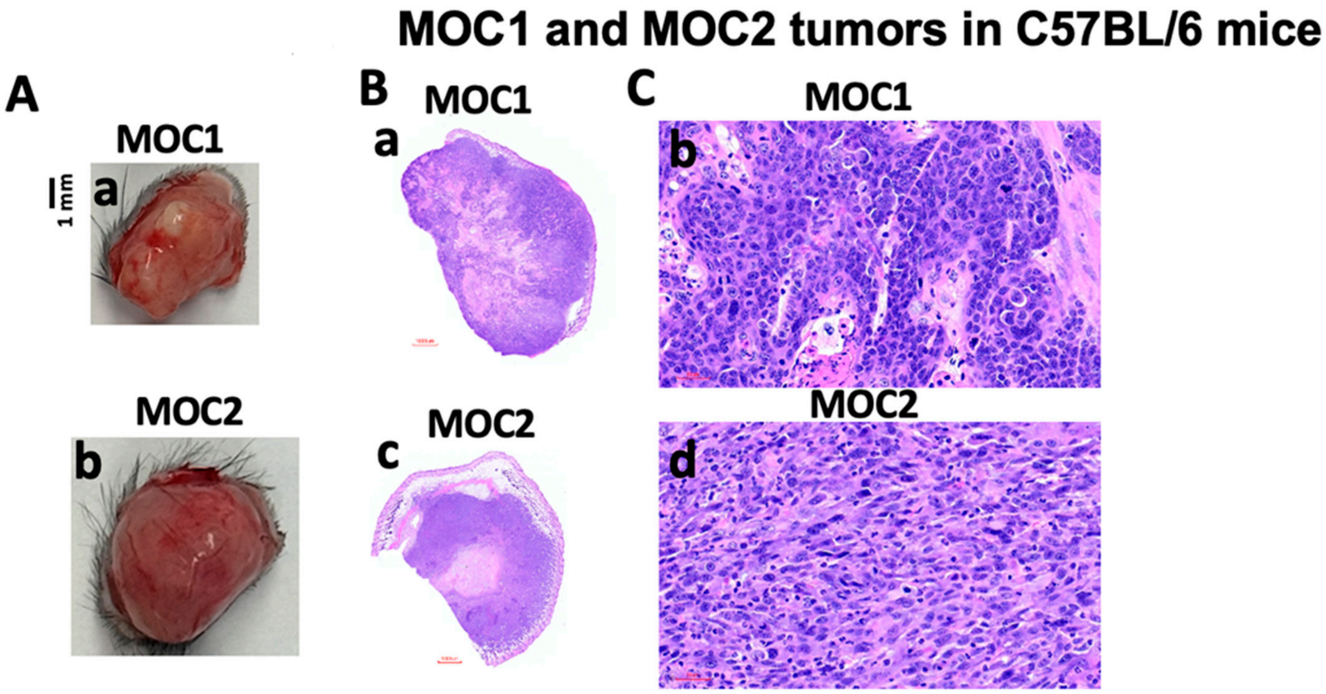

Figure S1. MOC1 and MOC2 tumors. (A) Gross view of representative MOC1 and MOC2 tumors dissected from C57BL/6 mice. **(B)** Images of representative H &E staining of MOC1 and MOC2 tumors. **(C)** Morphology of H&E staining of MOC1 and MOC2 tumors.

Supplementary Figure S2:

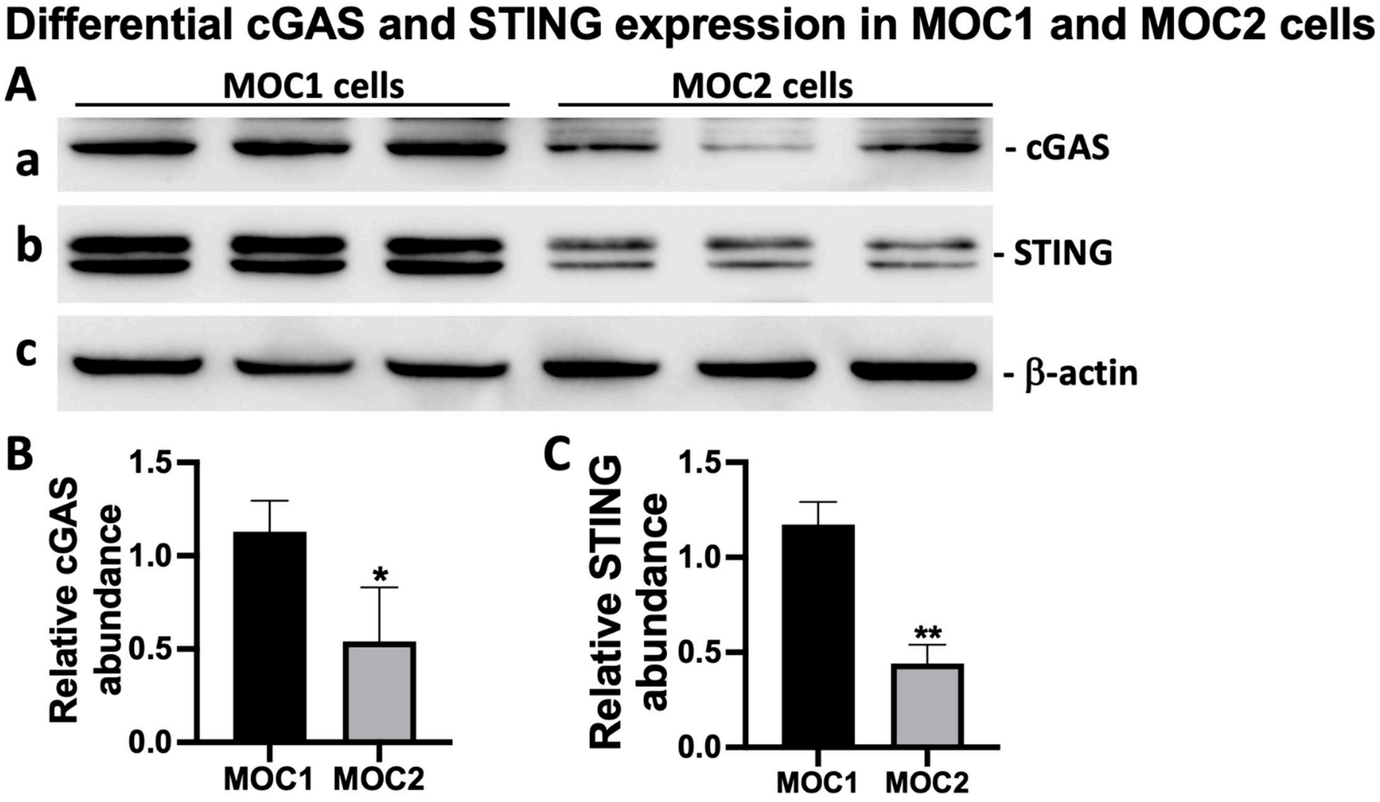

Figure S2. Expression of cGAS and STING in MOC1 or MOC2 cells. (A) Protein levels of cGAS, STING and β-actin in cultured MOC1 and MOC2 cells were determined by Western blot analysis. **(a)** cGAS protein level in MOC1 and MOC2 cells. **(b)** STING protein level in MOC1 and MOC2 cells. **(c)** β-actin protein level in MOC1 and MOC2 cells as controls. (**B**) Quantification of cGAS protein levels normalized to β-actin in MOC1 and MOC2 cells (n=3). (**C**) Quantification of STING protein levels normalized to β-actin in MOC1 and MOC2 cells (n=3).

Supplementary Figure S3:

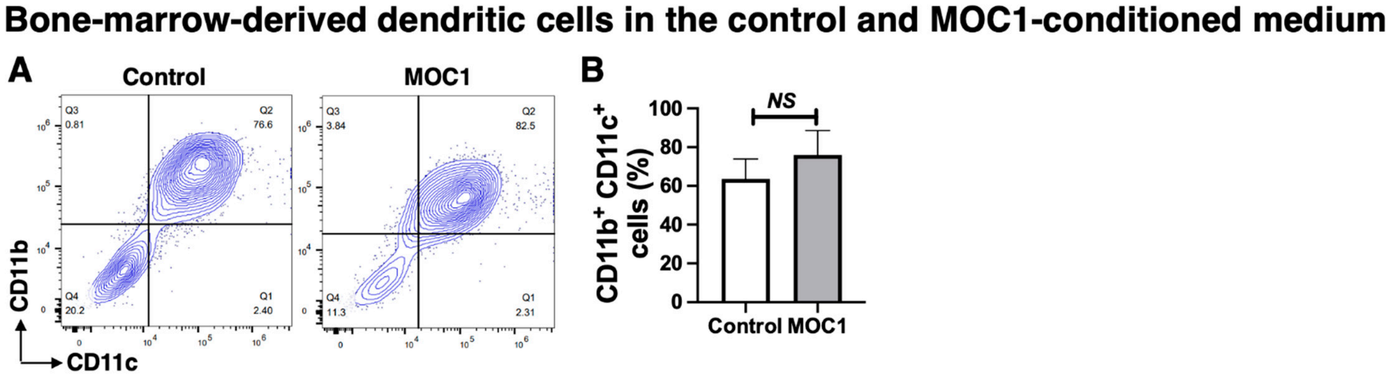

Figure S3. MOC1-conditioned medium does not reduce mouse bone marrow-derived dendritic cell differentiation. (A) Representative flow cytometry analysis of mouse bone marrow cell-derived $CD11b^{+}CD11c^{+}$ dendritic cells in control or MOC1-conditioned medium. **(B)** Quantitative analysis of $CD11b^{+}CD11c^{+}$ positive cells in control or MOC1-conditioned medium. *NS*: not significant.
