# Supplementary material for: Cancer Cell-Intrinsic Type I Interferon Signaling Promotes Antitumor Immunity in Head and Neck Squamous Cell Carcinoma
Source: Cancers (Basel). 2025 Apr 10;17(8):1279. doi: 10.3390/cancers17081279 (PMC12025670; doi:10.3390/cancers17081279)

Suppl. Figure S2 cGAS

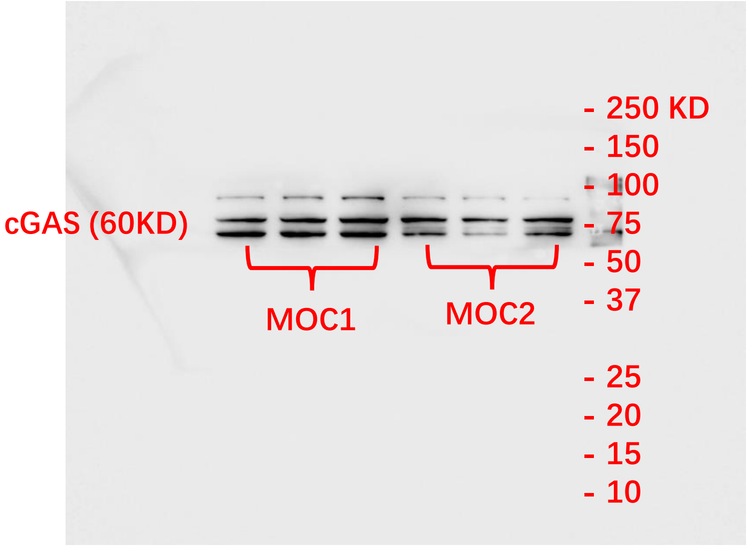

Suppl. Figure S2 STING

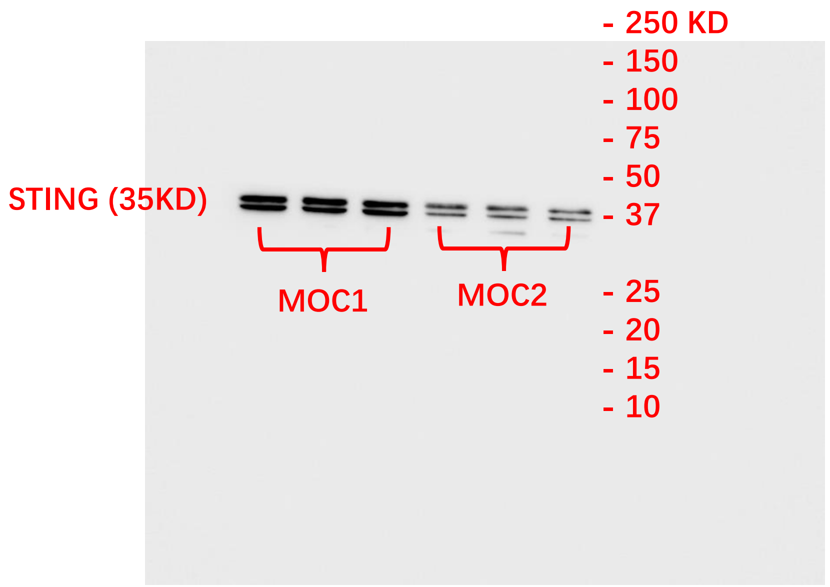

Suppl. Figure S2 Actin

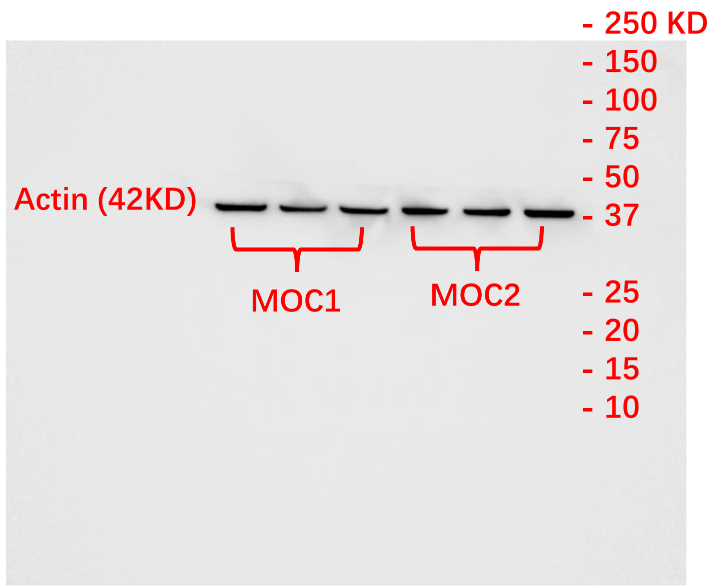

Figure 5Aa. GM-CSF protein levels in MOC2^{SIIN} cells expressing either vector control or exogenous GM-CSF

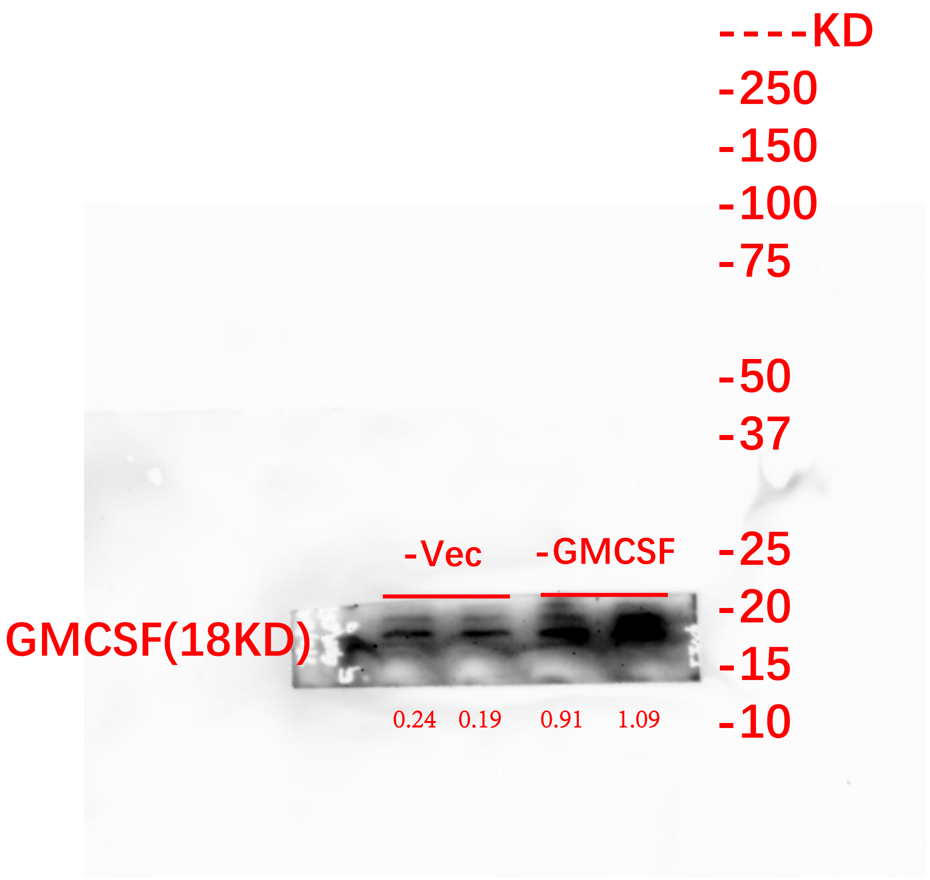

Figure 5Aa. β-Actin levels in MOC2^{SIIN} cells expressing either vector control or exogenous GM-CSF

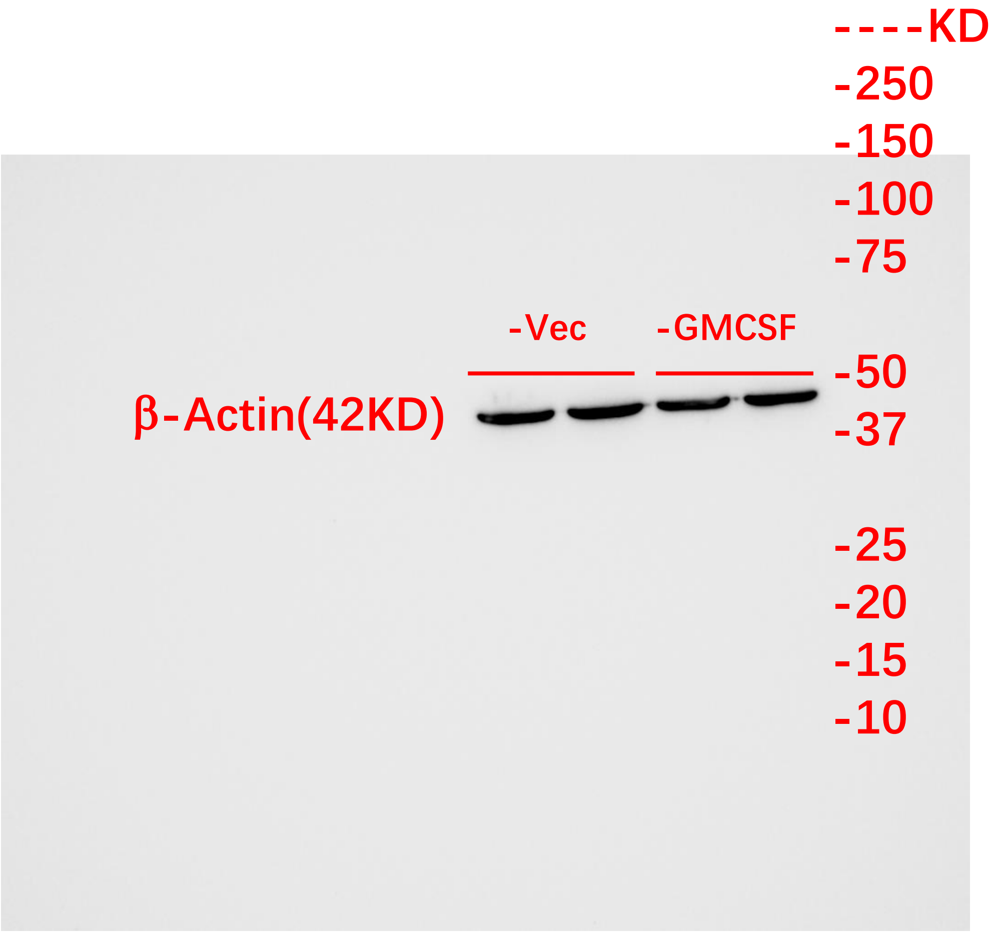

Supplement: Supplementary file 1 [file cancers-17-01279-s001.zip › The original blots for Figures 5 and S2.pdf]
